# Cardiac SR-Mitochondria Contacts—Impact on Cardiac Physiology and Mitochondrial Fitness

**DOI:** 10.3390/cells14221762

**Published:** 2025-11-11

**Authors:** Celia Fernandez-Sanz, Shey-Shing Sheu, Sergio De la Fuente

**Affiliations:** 1 Center for Translational Medicine, Department of Medicine, Sidney Kimmel Medical College, Thomas Jefferson University, Philadelphia, PA 19107, USA; 2 Instituto de Biología y Genética Molecular (IBGM), Departamento de Bioquímica y Biología Molecular y Fisiología, Facultad de Medicina, Universidad de Valladolid and Consejo Superior de Investigaciones Científicas (CSIC), Ramon y Cajal, 7, E-47005 Valladolid, Spain

**Keywords:** heart, MAMs, mitochondria, SR, bioenergetics, microdomains

## Abstract

In adult cardiomyocytes, within the Mitochondrial Associated Membranes (MAMs), the sarcoplasmic reticulum (SR) and mitochondria juxtapose each other, forming a unique and highly repetitive functional structure throughout the cells. These SR-mitochondria contact sites have emerged as critical structures that regulate various physiological processes, including lipid exchange, calcium (Ca^2+^) communication, control of excitation-contraction bioenergetics coupling, and reactive oxygen species (ROS) production. Over the years, several scientific studies have reported the accumulation of diverse proteins within these SR-mitochondria close contacts. Some proteins strategically accumulate in these areas to enhance their function, such as the mitochondrial Ca^2+^ uniporter, while others perform non-canonical roles, such as DRP1 acting as a bioenergetics regulator. The purpose of this review is to provide a comprehensive compilation of the proteins that have been reported to be enriched in cardiac MAMs. We aim to show how their positioning is crucial for proper cardiac physiology and fitness, as well as how mispositioning may contribute to cardiac diseases. Additionally, we will discuss the gaps in our understanding and identify the necessary components to fully comprehend physiological communication between the sarcoplasmic SR and mitochondria in cardiac tissue.

## 1. Introduction

The physiological relationship among cellular organelles is an aspect of cellular biology that has gained significant attention recently [1]. Within the cells, mitochondria are not isolated entities but rather dynamic organelles that actively communicate with other cellular components, including the endoplasmic reticulum (ER). The interaction between mitochondria and the ER occurs at specialized sites known as MAMs (Mitochondria Associated Membranes), where the membranes of these two organelles come into proximity.

MAMs serve as hubs for multiple signaling pathways crucial for cellular function and maintenance. For instance, they play a critical role in Ca^2+^ signaling by enabling the rapid and controlled transfer of Ca^2+^ between the ER and mitochondria. This close association allows for precise regulation of mitochondrial metabolism, ATP production, and cellular responses to stress and apoptosis [2,3].

Furthermore, MAMs are involved in lipid metabolism, facilitating the synthesis and transfer of lipids between the ER and mitochondria. This exchange is vital for maintaining cellular lipid balance and supporting phospholipids’ biosynthesis for mitochondrial membrane maintenance [4,5].

Recent research has also shed light on the role of MAMs in neurodegenerative diseases, metabolic disorders, and viral infections. Dysregulation of MAM function has been implicated in conditions such as Alzheimer’s disease, Parkinson’s disease, and diabetes, emphasizing the importance of understanding the intricacies of these specialized membrane contact sites [6,7].

MAMs in the heart are paramount due to their involvement in various crucial processes that impact cardiac function. One such process is Ca^2+^ signaling, essential for regulating heart muscle contraction and relaxation. The proximity of mitochondria and the sarcoplasmic reticulum (SR) at MAMs facilitates the rapid and precise exchange of Ca^2+^ between these organelles. This inter-organelle communication ensures tight control of Ca^2+^ levels, critical for maintaining proper heart rhythm and preventing pathological conditions such as arrhythmias [8].

Moreover, MAMs play a significant role in cardiac energy metabolism. The heart is a highly energy-demanding organ, relying primarily on mitochondrial oxidative phosphorylation to generate ATP, the cell’s primary energy source. MAMs facilitate the efficient exchange of lipids between the SR and mitochondria, ensuring a constant supply of fatty acids for beta-oxidation, the process by which fatty acids are broken down to produce ATP. This lipid transfer is crucial for sustaining the heart’s energy requirements and ensuring its optimal function [9].

MAMs have been implicated in regulating cellular stress responses in the heart. The SR serves as a reservoir of Ca^2+^ and is involved in the unfolded protein response (UPR), a mechanism activated under conditions of SR stress. The interaction between the SR and mitochondria at MAMs allows the transfer of signals and molecules involved in UPR, influencing cellular responses to stressors and protecting cardiac cells from damage [10].

Recent studies have also highlighted the role of MAMs in cardiovascular diseases, including heart failure, ischemia–reperfusion injury, and hypertrophy [11]. Dysregulation of MAM function has been linked to impaired Ca^2+^ handling, mitochondrial dysfunction, and oxidative stress, all of which contribute to the pathogenesis of these cardiac conditions [12,13]. Understanding the intricate workings of MAMs in the context of heart disease holds tremendous potential for developing novel therapeutic strategies to mitigate the detrimental effects of these conditions and improve patient outcomes.

## 2. Mitochondria-Associated Membranes: Discovery and Relevance

The physical relationship between the Sarco/endoplasmic reticulum (SR/ER) and mitochondria was first discovered in 1958, suggesting that the cooperation between these two organelles could be key for the conversion of carbon dioxide to bicarbonate and pH regulation [14]. Thirty years later, a cellular fraction known MAM was isolated for the first time from rat liver using differential centrifugation and a Percoll gradient from crude mitochondria [15]. One of the main characteristics of this specific mitochondrial fraction, referred to as Fraction-X by the authors, was its capability to synthesize serine-derived phospholipids. These activities, typically found in the ER fraction, were completely absent in the highly purified mitochondrial fraction. The so-called Fraction-X or MAM shared many similarities with the ER fraction, except that it sedimented with the mitochondria at 10,000× *g*. These results confirmed the existence of MAM in rat liver and its role in serine phospholipid synthesis and compartmentalization.

Several years later, pioneering studies by Rizzuto and colleagues demonstrated the existence of Ca^2+^ microdomains around ER-mitochondria subcellular regions in HeLa cells, emphasizing the importance of cell architecture and inter-organelle interaction in regulating Ca^2+^ signaling [16]. In this study, in vivo imaging showed that close appositions between the ER and mitochondria, or MAM, may represent the site where high Ca^2+^ concentration ([Ca^2+^]) microdomains are generated upon IP3-mediated Ca^2+^ release.

MAM’s functionality and structural characteristics have been intensely studied since its discovery. Confocal microscopy imaging using mitochondria- and ER-targeted spectral variants of green fluorescent protein allowed the visualization of the tethering structure connecting both organelles in mouse embryonic fibroblasts (MEFs) [17]. With this technical approach, the authors proposed an inter-organelle distance of less than 270 nm. Early electron microscopy studies revealed an average distance between the clusters of RyR2 in SR and mitochondria ranging from 37 to 270 nm in adult rat ventricular cardiomyocytes [18]. Electron tomography studies of adult rat liver revealed a tethering pattern of clusters of six or more units in which the intermembrane distance was as low as 6–15 nm [19]. These estimations align with later analysis of transmission electron micrographs of adult murine hearts, where the distance between the junctional SR and mitochondria averaged 15–20 nm [20,21].

In the heart, truncations, alterations, or modifications of the MAM environment have been demonstrated to be critical players in the pathophysiology of several conditions, with consequences on ATP availability, Ca^2+^ dynamics, and oxidative stress. Cardiomyocytes from failing hearts display reduced cytosolic Ca^2+^ transient amplitude, diminished SR Ca^2+^ content, and dysfunctional SERCA and RyR activities [22]. It has been suggested that these alterations may result from insufficient energy production and excessive ROS damage [23]. The pathophysiological mechanism underlying these deleterious effects could be the impairment of Ca^2+^ exchange between the SR and mitochondria [24]. Low mitochondrial Ca^2+^ uptake, secondary to defective SR-mitochondria communication in the MAM, could lead to a mismatch between energy demand and supply and to excessive oxidative damage due to insufficient regeneration of nicotinamide adenine dinucleotide phosphate (NAD(P)H) [20,25,26].

A defective MAM organization can also cause cardiac arrhythmias and sudden death as Ca^2+^-dependent arrhythmias, specifically atrial fibrillation, which are generally associated with instability of SR Ca^2+^ release in a pro-oxidative environment [27]. Similarly, sequential dysfunction of SR Ca^2+^ handling and mitochondrial function has been described in diabetic cardiomyopathy [28].

In the murine heart, the disruption of the SR-mitochondria spatial relationship occurring during aging is accompanied by defective mitochondrial Ca^2+^ uptake, and this effect has been associated with decreased NAD(P)H regeneration and increased mitochondrial ROS. Thus, an inadequate MAM environment underlies inefficient inter-organelle Ca^2+^ exchange, contributing to an energy demand/supply mismatch and oxidative stress in the aged heart [26].

This aging-related MAM disorganization in the heart is also associated with reduced tolerance to ischemia–reperfusion insults through mechanisms that involve increased oxidation of critical subunits of the FoF1 ATP synthase, higher incidences of mitochondrial permeability transition pore (mPTP) opening, and cell death [29]. Altogether, these observations support the existence of subcellular anatomical structures in which the SR and mitochondria are closely juxtaposed, playing a critical l role in cardiac physiology pathology.

## 3. Mitochondria-Associated Membranes Isolation from Murine Cardiac Tissue

All the previous findings highlight the importance of having a reliable and consistent method to isolate MAM from mammalian tissues. To help unravel the mechanism and function of MAM in cardiac tissue, we describe how to obtain highly purified MAM, mitochondria, and other subcellular fractions from murine hearts. It is important to note that the protocol can be easily modified to isolate MAM from other organs, tissues, or cell types [30].

**Figure 1:**
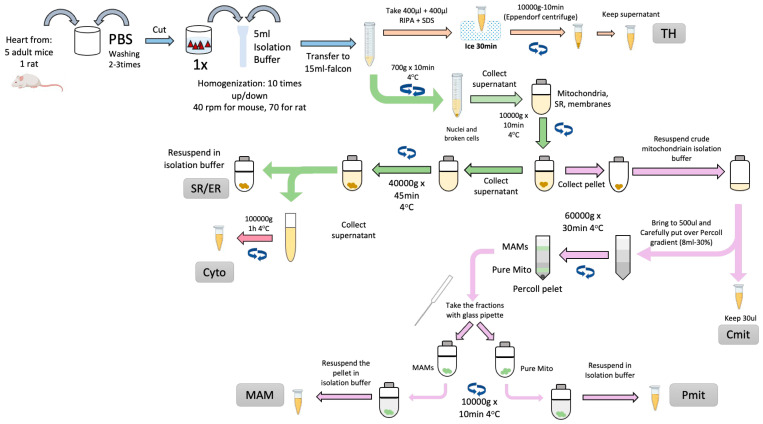
Scheme of MAM and cellular fractions isolation from cardiac tissue. The cellular fractions obtained are Total Homogenate (TH), Sarco/Endoplasmic Reticulum (SR/ER), Cytosol (Cyto), Crude Mitochondria (Cmit), Mitochondria Associated Membranes (MAM), and Pure Mitochondria (Pmit).

The procedure can be divided into two main sections. The first part consists of the obtention of crude mitochondria from the tissues. The protocol’s second part will include isolating the highly purified mitochondria and the MAM from the crude mitochondria. A comprehensive summary of the isolation of cardiac MAM and subcellular fraction is depicted in Figure 1.

Fresh cardiac ventricles are extracted, cleaned, minced, and homogenized using a Potter-Elvehjem PTFE pestle-glass tube (MERCK, Rockville, MD 20850, USA) at 40 rpm on ice-cold isolation buffer (in mmol/L): 10 HEPES acid, 225 mannitol, 75 sucrose, 0.1 EGTA, pH 7.4 with 10 Tris base, protease inhibitors).

Nuclei and other cell debris will be discarded in the pellet from an initial centrifugation step (750× *g* for 5 min, 4 °C). The supernatant, containing the rest of the cellular components, is collected and submitted to a 10,000× *g* centrifugation at 4 °C for 10 min. The resulting pellet, containing crude mitochondria, is collected and maintained on ice for further Percoll^®^ purification and MAM extraction. The supernatant was centrifuged at 40,000× *g*, 4 °C for 45 min to obtain the SR in the pellet. The supernatant will be cleared by ultracentrifugation at 100,000× *g*, 4 °C for 1 h, yielding the cytosolic fraction.

**Figure 2:**
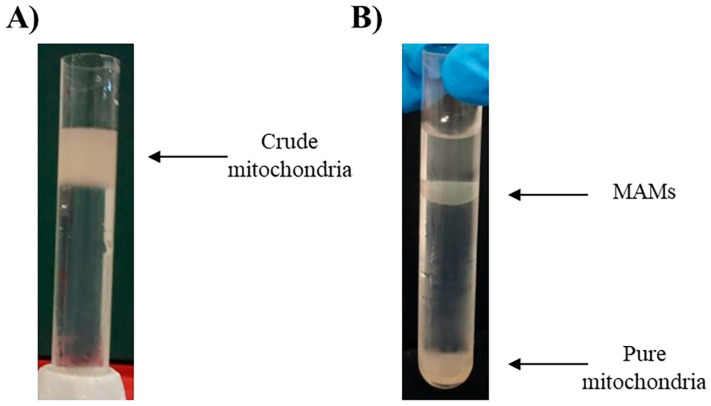
(**A**) Crude mitochondria over the 8 mL 30% Percoll^®^ gradient column. (**B**) Separated fractions of MAMs and pure mitochondria after ultracentrifugation using Percoll^®^ gradient [31].

The previously isolated crude mitochondrial fraction will be placed on an 8 mL 30% Percoll^®^ gradient column (MERCK, Rockville, MD 20850, USA) [31] (Figure 2A). The Percoll^®^ gradient column will be ultracentrifuged at 60,000× *g*, 4 °C for 30 min in a swinging rotor for the separation of pure mitochondria (heavy fraction) and MAM (light fraction). Both fractions (Figure 2B) can now be extracted from the column. The fractions can be easily cleaned from Percoll^®^ residues by washing them with 25 mL of isolation buffer followed by centrifugation at 12,000× *g* for 10 min at 4 °C. The pellets will contain pure mitochondria and MAM, respectively [26,32,33].

**Figure 3:**
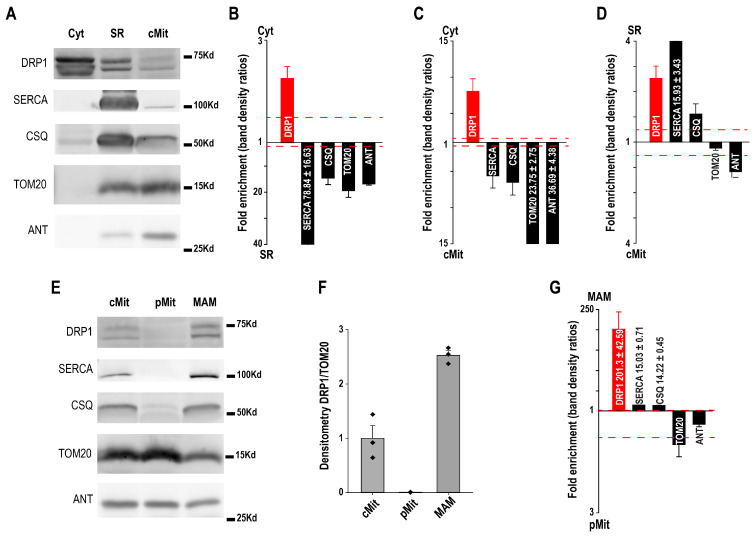
(**A**) Western blot of cytosol (Cyt), sarcoplasmic reticulum (SR), and crude mitochondria (cMit) fractions with SR (SERCA, CSQ) and mitochondrial (TOM20, ANT) markers. (**B**–**D**) Protein abundance comparisons across fractions. (**E**) Western blot of purified mitochondria (pMit) and mitochondria–SR associations (MAM), using the same markers. (**F**,**G**) DRP1 is enriched in MAM but not in pMit [30].

Western blotting was used to validate the purity of cardiac subcellular fractions and to examine the distribution of DRP1 (Figure 3). Representative blots show the expected enrichment of SERCA and CSQ in the SR fraction, and TOM20 and ANT in the mitochondrial fraction, confirming fraction identity, while also revealing some overlap consistent with SR–mitochondria contacts. DRP1 was detected in all fractions (Figure 3A).

Quantitative analysis indicated that DRP1 was more abundant in cytosol compared to both SR and mitochondria, while SR and mitochondrial markers displayed reciprocal enrichment (Figure 3B,C). Comparison of SR versus mitochondria revealed a relative enrichment of DRP1 in SR, supporting its association with SR membranes (Figure 3D).

After Percol purification crude mitochondria (cMit) were separated into purified mitochondria (pMit) and mitochondria–SR contact sites (MAM). Marker distribution confirmed pMit enrichment in mitochondrial proteins and MAM enrichment in SR proteins. DRP1 was almost absent from purified mitochondria but strongly detected in MAM. Densitometry confirmed higher DRP1/TOM20 ratios in MAM compared to both cMit and pMit. Quantitative comparisons showed a dramatic DRP1 enrichment in MAM relative to purified mitochondria, whereas classical SR markers (SERCA, CSQ) also tracked with MAM, further validating this fraction (Figure 3E–G).

A similar approach was used by our group to determine the sub-mitochondrial localization for the main influx and efflux Ca^2+^ pathways, the MCU and NCLX. The Western blot analysis for the cMit, pMit, and jSR fractions showed that the MCU is highly enriched in the jSR fraction compared to the pMit fraction, meaning that the MCU is specifically localized in those mitochondria that remain attached to the jSR after fractionation [34]. On the other hand, the main mitochondrial Ca^2+^ extrusion system, the NCLX, showed the opposite localization within cardiac mitochondria. Most of the NCLX seems to be present in the pMt while totally excluded from the jSR fraction, pointing out that NCLX is excluded from the mitochondrial area where the Ca^2+^ transporter from SR to mitochondria occurs. In this set of experiments, CSQ was used as an SR marker while Citrate Synthase was used as a mitochondrial marker [35].

Together, these data illustrate how Western blot can be used not only to confirm subcellular fraction purity through marker proteins, but also to reveal the preferential localization of DRP, MCU, and NCLX.

## 4. Relevant Proteins Reported to Be Localized at the Cardiac MAMs

Many proteins have been described to be present or physically tether the ER with the mitochondria. Several reviews compiled all the tethering complexes and proteins that are related to the MAMs in different tissues. However, these reviews will put the focus on those proteins that are present and have a physiological role in cardiac tissue, detailing their reported location and function at the MAMs and eventual physiopathology triggered by its mispositioning or ablation. Firstly, the tether complexes will be explained, and then proteins not involved in physical tether but reported to be enriched at the cardiac MAM will be described.

### 4.1. MFN2-MFN2 Complex

Mitofusin 2 (MFN2) is a protein primarily located in cells’ outer mitochondrial membrane (OMM). It belongs to the Mitofusin family of GTPases, which are involved in the regulation of mitochondrial fusion and morphology. MFN2 plays a critical role in maintaining the integrity of the mitochondrial network and regulating various cellular processes, including mitochondrial metabolism, Ca^2+^ signaling, apoptosis, and autophagy [36,37]. Therefore, MFN2 also maintains the structural and functional integrity of the mitochondrial network in cardiac cells. MFN2-mediated mitochondrial fusion ensures that mitochondria are well-distributed throughout the cardiomyocyte and can efficiently produce ATP, the energy source required for muscle contraction [38]. MFN2 is widely known to mediate mitochondrial fusion. However, its role as an SR-mitochondrial tether is still under debate [39].

MFN2 was first identified as a physical linker that brings the ER and mitochondria into close proximity. MFN2 is localized not only on the outer mitochondrial membrane (OMM) but also on a subset of the ER membrane facing mitochondria. Through homotypic (MFN2–MFN2) or heterotypic (MFN2–MFN1) interactions across the two organelles, MFN2 forms molecular bridges that maintain stable ER–mitochondria contacts. These contacts facilitate efficient Ca^2+^ transfer, lipid exchange, and metabolic coupling, which are critical for cardiomyocyte energetics and survival. Loss of MFN2 reduces the ER-mitochondria apposition, leading to diminished Ca^2+^ transfer from the sarcoplasmic reticulum (SR) to mitochondria, blunted oxidative phosphorylation, and energetic failure. This tethering model is supported by early studies [17] showing that MFN2 deficiency increased the distance between the ER and mitochondria and impaired interorganelle communication.

Subsequent studies introduced a contrasting model, proposing that MFN2 actually acts as a negative regulator—or spacer—of ER–mitochondria coupling. In this view, loss of MFN2 increases ER–mitochondrial proximity and Ca^2+^ transfer, potentially leading to Ca^2+^ overload, mitochondrial dysfunction, and apoptosis. The authors in [40] demonstrated that MFN2 ablation enhanced physical contacts between the two organelles and increased mitochondrial Ca^2+^ uptake in several cell types. Mechanistically, MFN2 may modulate the activity or positioning of other MAM tethers (e.g., VDAC1–IP_3_R–GRP75 complex or PDZD8) rather than serving as a direct structural link itself. Thus, MFN2 could function as a dynamic regulator that restrains excessive ER–mitochondria contact, preventing pathological Ca^2+^ flux under stress conditions.

The apparent contradiction—tether versus spacer—likely reflects cell type, metabolic state, and disease context. In energy-demanding tissues such as the heart, MFN2 appears to act predominantly as a functional tether, maintaining optimal ER–mitochondrial coupling necessary for ATP production and Ca^2+^ homeostasis. In contrast, in proliferative or stress-sensitive cells, MFN2 may serve as a regulatory spacer, preventing excessive contact that could trigger mitochondrial Ca^2+^ overload and apoptosis. Moreover, MFN2’s role is influenced by its post-translational modifications (e.g., phosphorylation, ubiquitination) and by interactions with other MAM components (such as PINK1/Parkin during mitophagy).

Despite the controversy of MFN2 functioning as a tether or spacer, its ablation led to several cardiac phenotypes. On one hand, Mfn2 deletion has been reported to protect against ischemia–reperfusion injury [41,42], but on the other, it may lead to cardiac hypertrophy and heart failure [43,44,45].

### 4.2. IP3Rs-GRP75-VDAC Complex

This complex has been extensively studied and encompasses three different proteins. IP3R (inositol 1,4,5-trisphosphate receptor) is a Ca^2+^ channel located on the endoplasmic reticulum (ER) membrane, responsible for releasing Ca^2+^ from the ER into the cytoplasm in response to the binding of IP3 (inositol 1,4,5-trisphosphate) [46]. GRP75 is a mitochondrial chaperone protein that assists in protein folding and transportation in the mitochondria [47]. VDAC is an integral membrane protein located on the outer mitochondrial membrane, forming a pore that allows the passage of ions, metabolites, and other molecules between the cytosol and mitochondria [48].

The IP3R-GRP75-VDAC complex forms a physical association between these three proteins. It has been proposed that GRP75 acts as a bridge between IP3R on the SR membrane and VDAC on the outer mitochondrial membrane, facilitating the transfer of Ca^2+^ ions from the ER to the mitochondria. This complex plays a role in regulating Ca^2+^ signaling and mitochondrial function, particularly in the context of cellular stress responses and apoptosis [49,50].

Similarly to MFN2, the disruption of the complex by inhibition of IP3R not only can protect cardiomyocytes against ischemia–reperfusion mediated by cyclophilin D but also decrease myocardial injury and heart failure mediated by less Ca^2+^ transference between SR to mitochondria [51,52,53].

### 4.3. VAPB-PTPIP51 Complex

The VAPB-PTPIP51 complex refers to the interaction between two proteins: VAPB (Vesicle-Associated Membrane Protein-Associated Protein B) and PTPIP51 (Protein Tyrosine Phosphatase-Interacting Protein). VAPB is an ER-resident transmembrane protein involved in multiple cellular processes, including lipid metabolism, ER-Golgi trafficking, and protein–protein interactions. It is primarily located in the ER, which functions as a scaffold protein [54].

PTPIP51 is a protein in the outer mitochondrial membrane and is involved in regulating Ca^2+^ signaling and mitochondrial function [55]. The VAPB-PTPIP51 complex was identified based on their physical interaction and co-localization at the MAMs.

This complex plays a role in regulating cellular processes such as lipid metabolism, ER-mitochondrial communication, and mitochondrial dynamics [56,57]. It has been implicated in neurodegenerative diseases, including amyotrophic lateral sclerosis (ALS) [58] and hereditary spastic paraplegia (HSP), where mutations in VAPB disrupt the regular interaction with PTPIP51, leading to cellular dysfunction and disease pathology.

Disruption of the complex through PTPIP51 deletion in cardiac tissue significantly reduces the cardiac damage upon ischemia–reperfusion due to the less Ca^2+^ transfer between the SR and mitochondria [59,60].

### 4.4. MCU

Ca^2+^ is involved in regulating mitochondrial metabolism by activating several enzymes involved in the tricarboxylic acid (TCA) cycle, the electron transport chain, and oxidative phosphorylation. The Mitochondrial Ca^2+^ uniporter (MCU)-mediated uptake of Ca^2+^ into the mitochondrial matrix is necessary to activate these enzymes and to stimulate mitochondrial respiration and ATP production [61]. Moreover, the MCU also regulates mitochondrial Ca^2+^ buffering, which helps to prevent calcium overload and mitochondrial dysfunction in cardiomyocytes [62]. There is no clear evidence that upon MCU absence, the cytosolic Ca^2+^ transients rise to higher levels due to the lack of mitochondrial Ca^2+^ buffering capacity. This fact might be caused by the higher activity of the plasma membrane Na^+^/Ca^2+^ exchanger that compensates for the MCU activity. However, this hypothesis has not been tested yet.

It was widely believed that MCU played a crucial role in adult cardiomyocytes because it helps to regulate intracellular Ca^2+^ levels and energy metabolism. However, several cardiac models lacking MCU showed that the presence of MCU is not required to maintain a proper bioenergetics-contraction coupling [63,64,65]. Cardiomyocytes require a significant amount of energy to contract and relax the heart; therefore, MCU-independent bioenergetics pathways are being explored to fully understand cardiac energy production and regulation [63,64].

In adult cardiomyocytes, studies have shown that the MCU is enriched in the junctional regions between the sarcoplasmic reticulum and the mitochondria, where they play a crucial role in Ca^2+^ signaling and energy production during contraction and relaxation of the heart [30]. The primary Ca^2+^ mitochondrial extrusion system in myocytes, the Na^+^/Ca^2+^ exchanger (NCLX), is mostly excluded from these areas where the MCU is. The bias distribution for both systems ensures an optimal Ca^2+^ signaling within single mitochondria and a mild membrane potential consumption [35,66]. Mispositioning on either of the methods could lead to a futile Ca^2+^ cycle and, therefore, mitochondrial damage [62].

### 4.5. Cyclophilin D

Cyclophilin D (CypD) is a peptidyl-prolyl cis-trans isomerase primarily located in the mitochondrial matrix of cells. It is a member of the cyclophilin family of proteins, which are known for their ability to bind to and modulate the activity of the immunosuppressant drug cyclosporin A [67].

CypD has been shown to play a critical role in regulating mitochondrial permeability transition pore (mPTP) opening, a process that involves the opening of a large, non-selective pore in the inner mitochondrial membrane. This process can lead to the release of pro-apoptotic factors and disruption of mitochondrial function, which has been implicated in various pathological conditions, including ischemia–reperfusion injury and neurodegenerative diseases [68].

In adult cardiomyocytes, CypD has been shown to play a critical role in regulating mPTP opening and mitochondrial function in response to various stressors, including ischemia–reperfusion injury and oxidative stress [69,70]. Inhibition of CypD has been shown to protect against mPTP opening and mitochondrial dysfunction.

It has been shown that CypD interacts with the complex IP3Rs-GRP75-VDAC at the MAM fraction [51]. When the CypD is removed from the SR-mitochondria interface, the interaction among the proteins present in the complex significantly decreases. This fact leads to less transfer between the SR and mitochondria, reducing the Ca^2+^ overload and mPTP opening chances. CypD may be a promising target for therapeutic intervention, particularly in cardiovascular diseases in this context.

### 4.6. FUNDC1

FUN14 domain-containing protein 1 (FUNDC1) is a transmembrane protein primarily located on mammalian cells’ outer mitochondrial membrane. It has been identified as a critical regulator of mitochondrial quality control and is involved in mitophagy, which is the selective degradation of damaged or dysfunctional mitochondria [71].

FUNDC1 plays a role in the initiation of mitophagy by recruiting autophagy machinery to the surface of damaged mitochondria. It does so by interacting with LC3, a protein that is involved in the formation of autophagosomes, and it has been shown to promote mitophagy in response to hypoxia, oxidative stress, and other cellular stressors [72].

It has been described that in adult cardiomyocytes [73], FUNDC1 is enriched at the contact sites between SR and mitochondria, helping the formation of MAMs. At the MAMs, FUNDC1 regulates the cellular Ca^2+^ homeostasis and dynamics to prevent heart diseases. The MAM regulation occurs through interactions with proteins such as calnexin and IP3Rs. Upon FUNDC1 ablation, adult cardiomyocytes present fewer MAM contacts and less IP3R and PACS-2 expression. Overexpression of FUNDC1 increased the number of MAMs in High-glucose in vitro models. Alterations in Ca^2+^ homeostasis, ROS production, and apoptosis caused by FUNDC1 ablation finally led to mitochondrial dysregulation, cardiac dysfunction, and heart failure [74].

### 4.7. GSK3β

Glycogen Synthase Kinase 3 Beta (GSK3β) is a serine/threonine protein kinase encoded by the GSK3β gene. GSK3β is a highly conserved enzyme that plays crucial roles in various cellular processes, including cell signaling, metabolism, gene expression, and cell cycle regulation [75]. GSK3β is involved in multiple signaling pathways, including the Wnt/β-catenin, insulin signaling, and mTOR (mammalian target of rapamycin) pathway. It phosphorylates a wide range of target proteins, thereby regulating their activity and function.

GSK3β is in various subcellular compartments within adult cardiomyocytes, including the cytoplasm, nucleus, and mitochondria. In particular, GSK3β has been shown to play an essential role in regulating mitochondrial function in these cells, including mitochondrial membrane potential, oxidative phosphorylation, and apoptosis [76]. The precise subcellular localization of GSK3B within the mitochondria is not well-defined.

However, it has been reported that a fraction of GSK3β is located at the SR-mitochondria interface, interacting with the IP3Rs [77]. Inhibition of GSK3B impaired the Ca^2+^ release and decreased the Ca^2+^ exchange between SR and Mitochondria in adult cardiomyocytes. Inhibition of GSK3β during the reperfusion period reduced the mitochondrial Ca^2+^ concentration and, therefore the sensitivity to myocardial apoptosis [77,78].

### 4.8. SIG-1R

Sigma 1 receptor (Sig-1R) is a transmembrane protein located in the ER membrane of cells. It is a multifunctional protein implicated in several cellular processes, including protein folding, lipid metabolism, ion channel regulation, and cell survival [79]. It has been found in many tissues, including the brain, heart, liver, and immune system [80].

Sig-1R is present at the MAMs [81,82]. Within the MAMs, Sig-1R works in conjunction with another chaperone, BiP. Sig-1R dissociates from BiP upon ligand stimulation and prolongs the mitochondrial Ca^2+^ entry via IP3R release. Sig-R1 KO mouse models show that, upon ablation, highly fused mitochondria and an abnormal cristae phenotype showed up, as well as significant cardiac fibrosis. Overall, Sig-1R loss of function leads to mitochondrial and cardiac dysfunction and heart failure [83].

### 4.9. DRP1

Dynamin-Related Protein 1 (DRP1) is a cytosolic GTPase protein involved in mitochondrial fission. It plays a critical role in dividing mitochondria into smaller fragments, essential for maintaining mitochondrial quality control, distribution, and function [84].

When activated, DRP1 is primarily located in the cytoplasm and translocated to the outer mitochondrial membrane. Once at the mitochondria, DRP1 assembles into a ring-like structure around the mitochondria, constricts, and facilitates mitochondrial division.

Various factors, including post-translational modifications, protein–protein interactions, and cellular signaling pathways, tightly regulate the fission process mediated by DRP1. Imbalances in mitochondrial fission and fusion dynamics, including aberrant DRP1 activity, have been associated with various diseases, including neurodegenerative disorders, cardiovascular diseases, and metabolic disorders [85].

DRP1 is recruited to the MAMs, where it interacts with proteins such as MFN2 and Fis1 (mitochondrial fission one protein) to initiate the fission process. The association between DRP1 and the mitochondrial-associated membranes is crucial for coordinating mitochondrial dynamics and ensuring proper distribution and wellness of mitochondria in cells. In adult cardiomyocytes, disruptions in proper mitochondrial fusion because of the absence of DRP1 lead to abnormal mitochondrial morphology and impaired mitochondrial function, contributing to the development of various cardiac diseases. In DRP1 KO cardiomyocytes, mitochondria showed decreased respiration and induced dilated cardiomyopathy [42,86].

### 4.10. Carbonic Anhydrase

When MAMs were first described in 1959, one of the hypotheses of such a specialized arrangement between the ER and the mitochondria in the pseudobranch cell was related to the production of carbonic anhydrase (CA) [14].

Since then, many isoforms of this enzyme, critical for the clearance of CO_2_ produced during mitochondrial respiration, have been described. The different CAs isoenzymes differ in their kinetic properties, tissue distribution, and subcellular localization [87].

The CA is especially crucial in the cardiac tissue, with the tricarboxylic acid cycle’s high mitochondrial CO_2_ production and the enzyme complex pyruvate dehydrogenase (PDH). Cardiac CAs catalyze CO_2_ hydration and the reverse reaction between H^+^ and HCO_3_^−^I ions. This biochemical reaction mediated by the CAs keeps CO_2_, HCO_3_^−^, and H^+^ at a chemical equilibrium even during increased workload, where the cardiac production of CO_2_ L^−1^·min^−1^ can increase several-fold compared with resting conditions [88].

It has been described that, in the heart, mitochondria are surrounded by an enriched CA domain. Interestingly, some of these CA appear to be associated with the SR at places of intimate contact between the two organelles. The CA microdomain helps to vent CO_2_ out of mitochondria, increasing the pH gradient across the mitochondrial inner membrane (ΔpHm), directly regulating cardiac bioenergetics [89].

### 4.11. PACS-1

Phosphofurin acidic cluster sorting protein 1 (PACS-1) is a multifunctional cytosolic sorting protein best known for its role in trafficking and localization of membrane proteins between the trans-Golgi network (TGN), endosomes, and the ER [90]. Structurally, PACS-1 contains an acidic cluster–binding domain that recognizes phosphorylated cargo proteins and a furin-binding domain, allowing it to act as an adaptor that links specific proteins to coat complexes and sorting machinery.

As a component of MAMs, PACS-1 plays a critical role in maintaining ER–mitochondria contact sites and Ca^2+^ signaling homeostasis. PACS-1 interacts with calnexin—a key ER chaperone enriched at MAMs—to regulate its localization between the bulk ER and the MAM subdomain [91]. Through this interaction, PACS-1 controls the proper positioning of calnexin at MAMs, which is essential for coordinating protein folding, lipid metabolism, and Ca^2+^ exchange between the ER and mitochondria. Loss or dysfunction of PACS-1 disrupts calnexin targeting, leading to altered ER–mitochondria tethering, impaired Ca^2+^ transfer, and mitochondrial stress

Beyond structural tethering, PACS-1 also influences cell survival signaling and mitochondrial dynamics. It modulates the interaction of apoptotic regulators (such as Bcl-2 family proteins) at MAMs and participates in stress responses that determine mitochondrial permeability and cell fate [92]. Dysregulation of PACS-1 has been implicated in neurodegenerative and metabolic disorders, and emerging evidence suggests that similar mechanisms may contribute to cardiac pathologies, where disturbed MAM integrity leads to energetic and Ca^2+^ handling deficits.

## 5. Conclusions

In the past few years, the “contactology” among organelles has gained more and more relevance within the cell biology sciences. The mitochondria-associated membranes stand out above the others, considering all the different cellular organelle interactions. MAMs usually refer to the ER/SR-mitochondrial contact points. Their function, composition, and physiopathology have been extensively studied, as mentioned in previous years. Virtually all cell types contain protein complexes that connect the ER/SR with mitochondria, facilitating critical cellular processes such as lipid synthesis and transfer, Ca^2+^ communication between the ER/SR and mitochondria, and mitochondrial dynamics. In adult cardiac tissue, the previously mentioned communication becomes a key player, especially regarding the Ca^2+^ SR-to-mitochondria transfer, since Ca^2+^ controls such essential processes as bioenergetic control, ROS generation, autophagy, or apoptosis. Therefore, it is not surprising that MAMs are critical regulators of cardiovascular physiological function.

The study of MAMs in adult cardiac tissue requires isolation and/or identification. The isolation of the MAM fraction relies on differential centrifugation, and its proteomic composition is often not fully understood. MAMs are those areas where SR and mitochondria are in close contact and frequently physically connected by a tether. Therefore, whenever MAMs are isolated, it implies the pull-down of mitochondrial membranes/proteins and SR membranes/proteins. Since no specific proteins are exclusively located at the MAMs, one should check for 50/50 enrichment on SR/Mitochondrial protein markers to verify a good MAM isolation. To compare, when talking about pure mitochondrial fraction, the enrichments should be about higher mitochondrial markers and lower for SR markers, or when talking about SR fraction, lower for mitochondrial markers and higher for SR markers. Note that obtaining a non-contaminated fraction is practically impossible and unavoidable.

MAM dysfunction contributes to cardiac diseases by disrupting the finely tuned communication between the endoplasmic/sarcoplasmic reticulum and mitochondria that maintains Ca^2+^ homeostasis, lipid metabolism, and bioenergetic balance. Under physiological conditions, the IP_3_R–GRP75–VDAC1–MCU complex at MAMs mediates controlled Ca^2+^ transfer to support mitochondrial ATP production required for cardiac contraction. However, excessive or reduced ER–mitochondrial coupling—often due to altered expression or localization of tethering proteins such as mitofusin-2 (MFN2), FUNDC1, or GRP75—leads to mitochondrial Ca^2+^ overload, reactive oxygen species (ROS) generation, mitochondrial permeability transition pore (mPTP) opening, and cardiomyocyte death. Concurrently, impaired MAM function disrupts phospholipid and cholesterol trafficking, compromises cristae integrity, and impairs oxidative phosphorylation, thereby precipitating energetic failure. Moreover, maladaptive MAM signaling induces endoplasmic reticulum stress, inflammasome activation, and fibrotic remodeling, all of which exacerbate heart failure and arrhythmogenesis. Collectively, MAM dysfunction drives a vicious cycle of Ca^2+^ dysregulation, mitochondrial damage, and inflammation that underlies myocardial ischemia/reperfusion injury, cardiomyopathy, and chronic heart failure [30,40].

MAM disruption contributes differently to acute ischemia–reperfusion (I/R) injury and chronic heart failure (HF), reflecting the distinct temporal and pathological demands on cardiomyocytes. In acute I/R injury, transient ischemia causes ATP depletion and cytosolic Ca^2+^ accumulation, while reperfusion suddenly restores oxygen, leading to excessive ER–mitochondrial Ca^2+^ transfer through the IP_3_R–GRP75–VDAC1–MCU complex. This uncontrolled Ca^2+^ influx into mitochondria triggers reactive oxygen species (ROS) generation, mitochondrial permeability transition pore (mPTP) opening, and necrotic/apoptotic cardiomyocyte death. MAM hypercoupling—often driven by upregulated or mislocalized tethering proteins like FUNDC1 or MFN2—exacerbates mitochondrial Ca^2+^ overload during reperfusion, amplifying oxidative stress and tissue injury. In contrast, chronic heart failure is marked by prolonged energetic stress and maladaptive remodeling, where MAM integrity is typically reduced or functionally impaired. Loss of proper ER–mitochondrial tethering (e.g., reduced MFN2 or disrupted lipid trafficking) diminishes the Ca^2+^ signaling needed to sustain mitochondrial oxidative phosphorylation, leading to ATP deficiency, accumulation of dysfunctional mitochondria, and impaired mitophagy [93,94].

Pharmacological and genetic strategies that restore or modulate mitochondria-associated membrane (MAM) integrity hold significant promise for treating cardiac diseases. In acute ischemia–reperfusion (I/R) injury, excessive ER–mitochondrial Ca^2+^ transfer via the IP_3_R–GRP75–VDAC1–MCU complex leads to mitochondrial Ca^2+^ overload, ROS production, and mPTP opening, driving cardiomyocyte death. Targeting this axis with IP_3_R or MCU inhibitors, GSK3β inhibitors, or ER stress modulators can reduce mitochondrial injury and infarct size. In contrast, chronic heart failure (HF) involves MAM uncoupling due to reduced expression or dysfunction of tethering proteins such as mitofusin-2 (MFN2) and FUNDC1, impairing Ca^2+^ signaling, mitophagy, and bioenergetics. Restoring MAM function through MFN2 overexpression, CRISPR correction, or small-molecule MFN2 activators improves mitochondrial quality control and ATP production. Agents like melatonin and metformin also stabilize MAMs indirectly by reducing ER stress and oxidative damage. Importantly, therapies must be context-specific: transient MAM suppression protects against Ca^2+^ overload in I/R, while MAM reinforcement supports energy homeostasis and survival in chronic HF. Collectively, MAMs represent a critical and druggable hub linking Ca^2+^ handling, metabolism, and cardiomyocyte fate [11,95].

This review summarizes the methodology currently used for MAM isolation, as well as going through most of the proteins located or reported to be enriched at the MAMs in adult cardiac tissue. Adult cardiomyocytes display a highly specialized cytoarchitecture in which mitochondria are regularly interspersed between myofibrils and tightly associated with the junctional sarcoplasmic reticulum (SR) at mitochondria-associated membranes (MAMs). This repetitive organization ensures an efficient excitation–contraction–bioenergetics coupling, where calcium fluxes and ATP production are spatially and temporally matched to the mechanical demands of the heartbeat. Within this unique architecture, key proteins such as the MCU and DRP1 are preferentially localized at the MAMs as reported by our group [30,34]. MCU enrichment at these sites allows mitochondria to sense local calcium microdomains generated by SR release, thereby stimulating oxidative metabolism precisely where and when energy is needed. Conversely, Drp1 is recruited to MAMs to coordinate mitochondrial fission in close alignment with SR-mitochondria tethering, ensuring the maintenance of mitochondrial quality, distribution, and adaptability in the energy-demanding cardiac environment. Thus, the positioning of MCU and Drp1 at MAMs reflects how cardiomyocyte architecture dictates protein localization to safeguard both bioenergetics and mitochondrial dynamics.

Ablation or mispositioning of most of the proteins mentioned in the present review frequently leads to cardiac phenotypes such as ischemia–reperfusion injuries, cardiomyopathies, or heart failure. Often, these diseases are directly related to SR and mitochondria miscommunication, leading to unpaired Ca^2+^ transfer, defective bioenergetics regulation, and apoptosis events.

## Data Availability

The original contributions presented in this study are included in the article. Further inquiries can be directed to the corresponding authors.
